# Experimental and Numerical Simulation to Study the Reduction of Welding Residual Stress by Ultrasonic Impact Treatment

**DOI:** 10.3390/ma13040837

**Published:** 2020-02-12

**Authors:** Jianfei Chen, Jingyu Chu, Wenchun Jiang, Bin Yao, Fan Zhou, Zhenbo Wang, Pengcheng Zhao

**Affiliations:** 1 School of New Energy, China University of Petroleum (East China), Qingdao 266580, China; chenjianfei121.slyt@sinopec.com (J.C.); jnchujingyu@126.com (J.C.); bestyaobin@163.com (B.Y.); zhoufanyx@126.com (F.Z.); wangzhb@upc.edu.cn (Z.W.); 2 Technology Inspection Center of Shengli Oil Field China Petroleum & Chemical Corporation, Dongying 257000, China; 3 College of Electromechanical Engineering, Qingdao University of Science and Technology, Qingdao 266061, China

**Keywords:** welding residual stress, ultrasonic impact treatment, X-ray diffraction, indentation strain method, finite element simulation

## Abstract

In this study, the effects of ultrasonic impact treatment (UIT) on the residual stress in a repair welding joint are investigated by experimental and finite element methods. A three-dimensional numerical analysis approach including a thermomechanical-coupled welding simulation and dynamic elastic-plastic UIT simulation is developed, which has been validated by X-ray diffraction measurement and indentation strain method. The results show that longitudinal residual stresses basically turned into the small tensile stress state from the large tensile stress state, and transverse residual stresses have mainly turned into compressive stresses from large tensile stress after the UIT. In the thickness direction, the average decrease of longitudinal residual stress is 259.9 MPa, which is larger than the 149.1 MPa of transverse residual stress. The calculated residual stress distribution after the UIT of the thin plate is compared with that of the thick plate in the literature, with the results showing the stress accumulation layer inside the thick plate. The simulation results show that the elastic strains are decreased slightly and the equivalent plastic strain is increased markedly after UIT, which explains the mechanism of residual stress relaxation.

## 1. Introduction

Thin 304 stainless steel plates are widely used in pipelines, automobiles, heat exchangers, etc. Damaged or worn parts are always generated in these structures, which need repair welding procedures to restore their working capability [1,2,3]. However, welding residual stresses are inevitably generated due to the nonuniform temperature distribution during repair welding. The existence of tensile residual stresses can especially decrease the load capacity and fatigue life, and increase the risk of stress corrosion cracking [4,5]. Therefore, it is significant to ensure the safety of welding structures by decreasing the harmful tensile residual stresses and to appropriately generate beneficial compressive residual stresses.

There are many post-weld methods mature in decreasing the welding residual stress, such as post-weld heat treatment [6], shot peening [7], post-weld rolling methods [8], water jet peening [9], etc. Some methods that are used before and during welding can also play an important role in decreasing welding residual stress, such as using proper groove distances [10], welding with low transformation temperature consumables [11], a preheating process [2], ultrasonic vibration [12,13,14], etc. However, compared with the above treatments, ultrasonic impact treatment (UIT) has superiority in reducing residual stresses and improving fatigue strength. Meanwhile, UIT has the effect of refining metal grains of surface layers, creating nanocrystalline layers in the surface, and increasing the hardness and wear resistance of material surfaces [15]. UIT is a cold-work surface treatment that uses the periodic impacts of high-frequency pins on the surface to reduce welding residual stresses and improve the mechanical properties. The ultrasonic transducer translates electrical signals generated by the ultrasonic generator into harmonic oscillations, and the waveguide amplifies mechanical vibrations and drives the pin to impact the treated surface [16,17]. When the pin contacts with the component surface, the focused energy with a high frequency is transferred to the component, which changes the original stress field, eliminates the tensile residual stresses, and generates a compressive residual stress layer. In addition, UIT could prolong the fatigue life and improve the fatigue resistance even in the corrosive and underwater environment [18,19,20]. Besides, owing to the advantages of easy operation and strong adaptability, UIT is extensively used in the aerospace, automotive, and power industries [21]. Cheng et al. [22] found that the UIT had a more stable impact pressure and a longer effective impact time compared with ultrasonic shot peening, so it produced a larger degree and a deeper layer of compressive residual stresses. Statnikov et al. [17] compared the improvement in fatigue strength on the welding joints treated by hammer peening, shot peening, TIG (Tungsten Inert Gas Welding) dressing and TIG dressing with subsequent UIT. The UIT could be more effective in increasing the fatigue strength by correctly choosing the conditions. Suominen et al. [23] have studied the improvement of residual stresses and fatigue strength in welding components treated by the UIT and laser remelting method with low-transformation temperature filler material. The experimental results showed that the effect of UIT on residual stresses and fatigue strength was stronger. Therefore, UIT has been successfully applied in various industrial structures and would have a greater prospect in the post-welding treatment.

Some research has focused on the UIT technology to decrease the welding residual stresses by using a finite element method (FEM) and experimental methods in recent years. Guo et al. [21] proposed two three-dimensional numerical models (single-impact and two-impact) to study the influence of the controlled UIT parameters on the residual stresses. They found that the increase of the pin initial velocity, the pin diameter and length led to a nonlinear increase in the depth of penetration, as well as the magnitude of the maximum compressive residual stresses. Hu et al. [24] simulated the successive impacts of five impact rows with a speed-based impact model. They pointed out that the continuous impact process during UIT was not a simple overlay of the multiple one-impact, in which it made the plastic deformation more homogeneous and reduced the maximum compressive residual stresses below the previous impact. They discussed the residual stresses generated by UIT, but did not consider the welding process. Liu et al. [25] used FEM and the contour method to investigate the exterior and internal residual stress variations in a high-strength steel welding joint after UIT. They revealed that the UIT resulted in lower residual stresses in the depths of 7–10 mm below the surface and generated a 4 mm-deep compressive residual stress layer.

Although some researches have been done on UIT, the accuracy of simulation and the relaxation mechanism of welding residual stress still needs further study. Furthermore, few studies paid attention to the effect of UIT on residual stress in the repair welding joint. In addition, the mechanism of stress relaxation by UIT is not clear. In this paper, a finite element model has been developed to decrease the welding residual stresses by UIT, which has been validated by X-ray diffraction (XRD) measurement and indentation strain method. Moreover, the mechanism of stress relaxation is discussed from macroscopic and microscopic perspective.

## 2. Materials and Experiments

### 2.1. Sample Preparation

**Figure 1:**
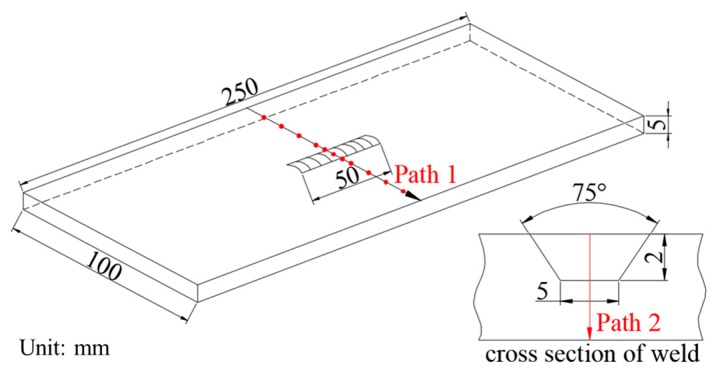
Dimensions of the repair welding sample.

Figure 1 shows a sketch of the repair welding sample with the single-pass welding. The dimensions of the base metal are 250 mm (length) × 10 mm (width) × 5 mm (thickness). The parent metal and filler wire are 304 stainless steel and A102, whose chemical compositions were tested by a Q4 Tasman direct reading spectrometer (BRUKER Company, Karlsruhe, Germany) as shown in Table 1. The chosen welding method is manual arc welding. The repair length, width, and depth are 50 mm, 5 mm and 2 mm, respectively. The weld groove angle is 75°. The electrode diameter is 4 mm. The arc current, voltage and welding speed are 110 A, 20 V and 3 mm/s, respectively. After XRD measurements of the as-welded residual stress, the UIT was employed to treat the welding region, heat affected zone (HAZ) and parent metal on the top surface. The impact pin has a cylindrical flat head with the diameter of 20 mm and length of 45 mm. The impact amplitude and frequency are 20 μm and 20 kHz, respectively.

### 2.2. X-ray Diffraction Measurement of Residual Stress

The residual stresses are measured by the XRD method, which is a nondestructive measurement technique [26]. The sin^2^*Ψ* method of XRD is applicable for the plane stress conditions, and is therefore used to measure residual stresses on the surface.

The theory of XRD method is based on Bragg’s law:

(1) λ=2dsinθ

where *λ* is the wavelength, *d* is the interatomic lattice spacing, and 2*θ* is the diffraction angle. The lattice strain for reflection (*hkl*) is determined by the shift of interatomic lattice spacing according to Equation (2) [27]:

(2) $$\varepsilon_{\psi }^{\left(hkl\right)}=\left(d_{\psi }^{\left(hkl\right)}-d_{0}\right)/d_{0}$$

where *ψ* is the orientation angle between normal lines of crystal surface and specimen. *d*_0_ is the lattice spacing in stress-free sample, and $d_{\psi }^{\left(hkl\right)}$ is lattice spacing in the welding sample. Considering the biaxial stresses in plane stress condition, the Equation (3) enables the calculation of strain at the orientation (*φ*, *Ψ*) [27]:

(3) $$\varepsilon_{\varphi ,\psi }=[\frac{1+\nu }{E}]\sigma_{\varphi }\sin^{2}\psi -(\frac{\nu }{E})\left(\sigma_{1}+\sigma_{2}\right)$$

where *ν* and *E* are the Poisson ratio and elastic constant, respectively. *σ*_1_ and *σ*_2_ are the first and second principal stress, respectively. *φ* stands for the orientation angle between normal line of crystal surface and *σ*_1_, *σ*_φ_ stands for the stress at orientation *φ* [27].

The stress could be obtained by [27]:

(4) $$\sigma_{\psi }=[-\frac{1}{2}\mathrm{ctg}\theta_{0}(\frac{E}{1+v})]\left(\frac{\partial 2\theta_{\varphi ,\psi }}{\partial \sin^{2}\psi }\right)$$

The diffractometer that was used is the X-350A, and the main parameters are listed in Table 2. Before measurements, the rough surfaces are ground and polished to be smooth for ensuring test quality. The measurement points are located in 0, 5, 10, 20, 30 and 40 mm away from the weld center, respectively, as shown in Figure 1.

### 2.3. Indentation Strain Measurement of Residual Stress

Indentation strain measurement was also conducted to measure the residual stress before and after UIT. The measurement positions are the same with those used in the X-ray diffraction measurement. Through applying an impact indentation load, the welding residual stress will be relaxed. Therefore, a strain increment occurs and can be detected by the strain gage [28]. The impact indentation measurement was carried out according to China National Standard GB/T 24179-2009. It can be assumed that the principal stresses are parallel and normal to the welding direction, namely longitudinal and transverse stress, which can be calculated as Equations (5) and (6) [28]:

(5) $$\sigma_{1}=\frac{E}{1-v^{2}}(\varepsilon_{e1}+v\varepsilon_{e2})$$

(6) $$\sigma_{2}=\frac{E}{1-v^{2}}(\varepsilon_{e2}+v\varepsilon_{e1})$$

where *σ*_1_ and *σ*_2_ mean longitudinal and transverse stress, respectively. *ε*_e1_ and *ε*_e2_ are the corresponding elastic strain.

## 3. Finite Element Simulation

**Figure 2:**
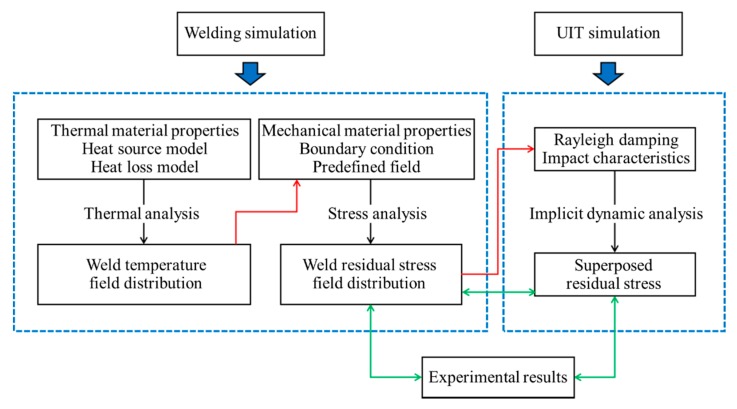
Flow chart of finite element simulation.

**Figure 3:**
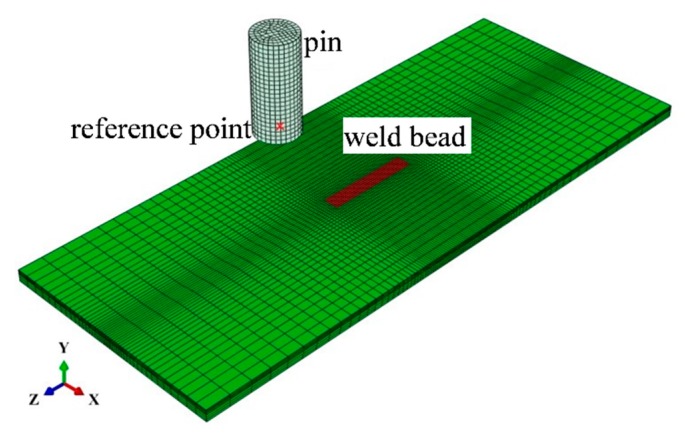
The finite element meshing.

A three-dimensional simulation procedure is carried out including the sequentially coupled thermomechanical welding simulation and the implicit dynamic simulation of the UIT process. An overview of the modelling procedure is shown in Figure 2. The finite element meshing is shown in Figure 3, which consists of 35,148 nodes and 29,904 elements. According to the mesh independent verification, the mesh with 1 mm × 0.5 mm × 1.5 mm near the weld is selected.

### 3.1. Simulation of Weld Temperature

The used heat source is the double ellipsoidal heat distribution model as proposed by Goldak [29]. It is a volume distribution heat source described by two semi ellipsoids of different sizes at the front and rear (Equations (7) and (8)) [30]:

(7) $$q_{f}\left(x,y,z\right)=\frac{6\sqrt{3}\left(f_{f}\eta IU\right)}{abc\pi \sqrt{\pi }}\exp\left(-\frac{3x^{2}}{a^{2}}-\frac{3y^{2}}{b^{2}}-\frac{3z^{2}}{c^{2}}\right)$$

(8) $$q_{r}\left(x,y,z\right)=\frac{6\sqrt{3}\left(f_{r}\eta IU\right)}{abd\pi \sqrt{\pi }}\exp\left(-\frac{3x^{2}}{a^{2}}-\frac{3y^{2}}{b^{2}}-\frac{3z^{2}}{d^{2}}\right)$$

where *f*_f_ and *f*_r_ are the parameters, which show the heat input fractions in the front and rear half ellipsoid bodies. Regulate that *f*_f_ + *f*_r_ = 2, in which *f*_f_ and *f*_r_ are assumed to be 1.4 and 0.6. The hypothesis is based on the fact that the temperature gradient in the front part is steeper than in the rear part, due to the movement of heat source. *a*, *b*, *c*, and *d* are the half-axis parameters of the semi ellipsoids, which are adjusted to get the desired weld pool size. *I* and *U* are current and voltage, which was determined during the experiment; *η* is assumed to be 0.7. They are the parameters that define the heat source energy. The moving heat source model is defined by a user subroutine DFLUX, which is compiled by FORTRAN language.

**Figure 4:**
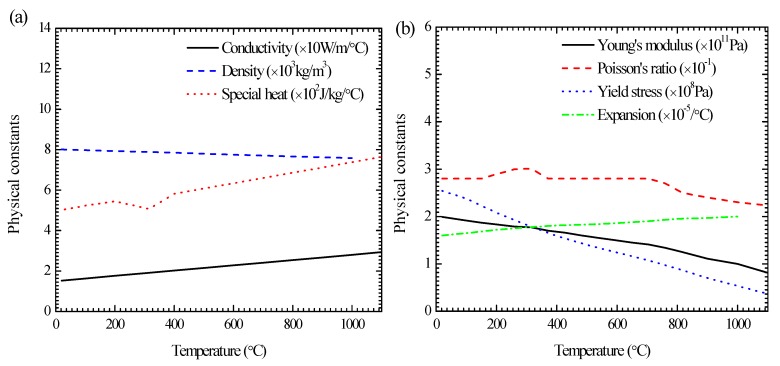
Temperature-dependent thermal properties (**a**) and mechanical properties (**b**) of 304 steel.

The related material properties are the temperature-dependent conductivity, density, specific heat and the latent heat, and their values are represented by lines in Figure 4a. The specific heat indicates the ability of heat absorption or heat dissipation, which is applied to calculate the thermal effect of molten pool solidification. The effect of phase transformation is considered by the latent heat. The heat loss model is created by comprehensively considering the thermal convection and the thermal radiation. In the simulation, the side faces of the patch plate are considered to be adiabatic. The boundary conditions of heat conduction are applied on the top and bottom surface. In order to fully dissipate heat, the cooling time is set to be 8000 s. The radiating efficiency is controlled by the total temperature-dependent heat transfer coefficient *h*, which is calculated by [31]:

(9) $$h=\frac{\varepsilon \sigma \left(T_{\mathrm{cur}}^{4}-T_{o}^{4}\right)}{T_{\mathrm{cur}}-T_{o}}+h_{c}$$

where ε is the emissivity defined as 0.85. The Stefan–Boltzmann constant *σ* is 5.669 × 10^−8^ W/m^2^K^4^. The current temperature *T*_cur_ is determined on computing process. The ambient temperature *T*_o_ is set to 20 °C. The absolute zero is inputted to –273.15 °C, which is applied to unit conversion in the calculating process. The convection coefficient *h*_c_ is defined as 15 W/m^2^K.

### 3.2. Simulation of Welding Residual Stress

During the stress analysis, the elastic parameters, the plastic parameters and the expansion coefficient need to be set. They are considered to be temperature-dependent as shown in Figure 4b. In this study, the solid-state phase transformation has not been considered in the welding process. Therefore, the welding residual stress is calculated by thermal elasto-plastic analysis. The total strain could be decomposed into elastic strain $\varepsilon_{e}$, plastic strain $\varepsilon_{p}$, and thermal strain $\varepsilon_{th}$ [32]:

(10) $$\varepsilon =\varepsilon_{e}+\varepsilon_{p}+\varepsilon_{\mathrm{th}}$$

The elastic strain follows the isotropic Hooke law with elastic parameters including Young’s modulus and Poisson’s ratio. The plastic strain obeys the Von Mises yield criterion represented by Equation (11) and isotropic hardening model with plastic parameters including yield stress and plastic strain:

(11) $${\left(\sigma_{1}-\sigma_{2}\right)}^{2}+{\left(\sigma_{2}-\sigma_{3}\right)}^{2}+{\left(\sigma_{3}-\sigma_{1}\right)}^{2}=2\sigma_{s}^{2}=6K^{2}$$

where *σ*_s_ is the yield point of material, and *K* is the shear yield strength of material. The thermal strain is calculated by thermal expansion coefficient *α*.

The model needs to be updated from the following three aspects: mesh, boundary condition and predefined field. Firstly, the stress simulation requires the same mesh as the temperature simulation, except the element type. The thermal unit needs to be changed to structural unit. Secondly, the boundary condition is added to the mechanical model, which limits the freedom of partial nodes. Thirdly, the temperature field obtained from the weld temperature simulation is input as predefined field to the welding residual stress simulation.

### 3.3. Simulation of UIT

**Figure 5:**
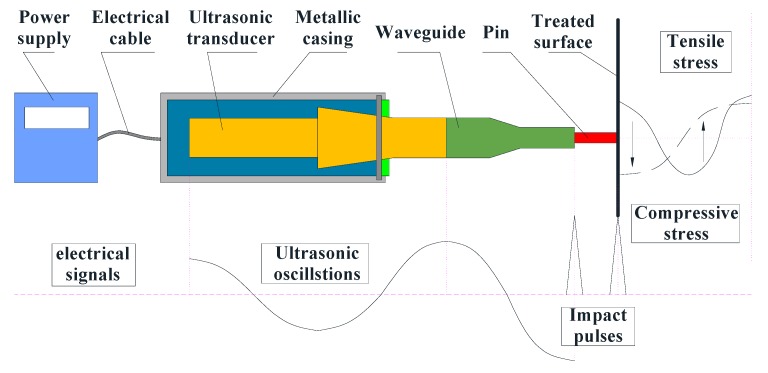
Schematic diagram of ultrasonic impact treatment (UIT).

**Figure 6:**
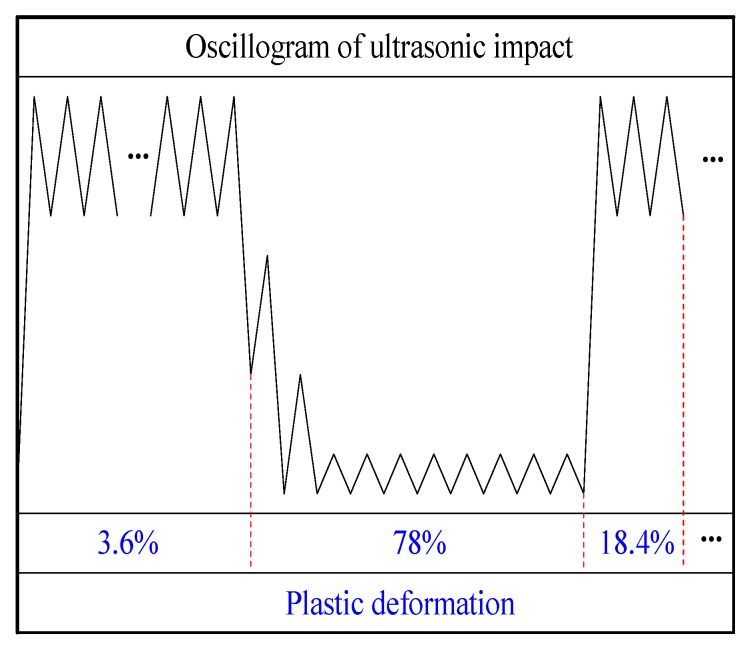
Relation between ultrasonic impact waveform and plastic deformation generation.

Figure 5 shows the UIT process based on the conversion of electrical signals into impact pulses. A sinusoidal electrical signal generated by an ultrasonic generator is transmitted from electric cable to an ultrasonic transducer. The conversion of electric energy to mechanical energy shown as ultrasonic oscillations is performed by an ultrasonic transducer. The mechanical vibrations are amplified by a waveguide. A pin moves freely between the waveguide and the treated surface as well as impacts on the treated surface, which implements the transformation from impact pulses to force pulses. Statnikov et al. [17] divided the ultrasonic impact into three stages: the frequency increase and plastic deformation saturation stage contributed only 3.6% of the total plastic deformation; the reboundless impact and stress wave propagation stage initiated as much as 78% of that deformation, and the frequency reduction and continuous plastic deformation stage. They also found that the energy utilization rate of UIT is at least 75% and is far higher than 25% of ultrasonic peening treatment. Yuan [31] pointed out the distinctive aspect of UIT and simplified the UIT model proposed by Statnikov [17] in the numerical simulation. Based on the work by Yuan [31] and Statnikov [17], it proposed the corresponding relationship between the simplified oscillogram and plastic deformation generation, as shown in Figure 6.

Because of the complexity of the UIT process, the following assumptions are made: (1) Since the deformation of the pin is much less than the treated plate, the pin is regarded as a rigid body and the impacted plate is considered as an elastoplastic material with nonlinear kinematic hardening model. The point on the cylindrical pin is defined as reference point (shown in Figure 3), at which the movement characteristics are set. (2) The output of the device is stable, showing that the vibration frequency and amplitude of the pin is constant that do not vary with time and position. The vibration mode of the pin as an important condition is subordinated to sine wave, as shown in Equation (12) [31]:

(12) x(t)=Asin(2πft)

where *A* is the amplitude, *f* is the frequency, and *t* is the time. According to the experiment, *A* is 20 μm, *f* is 20 kHz. According to the hypotheses, the high-frequency sine wave is replaced by a linear wave with the same amplitude and frequency, which simplifies the calculation greatly. (3) The impact of the pin on the treated surface is vertical impact, so the contact type is “hard” contact, and the load is pressure and there is no shear force.

**Figure 7:**
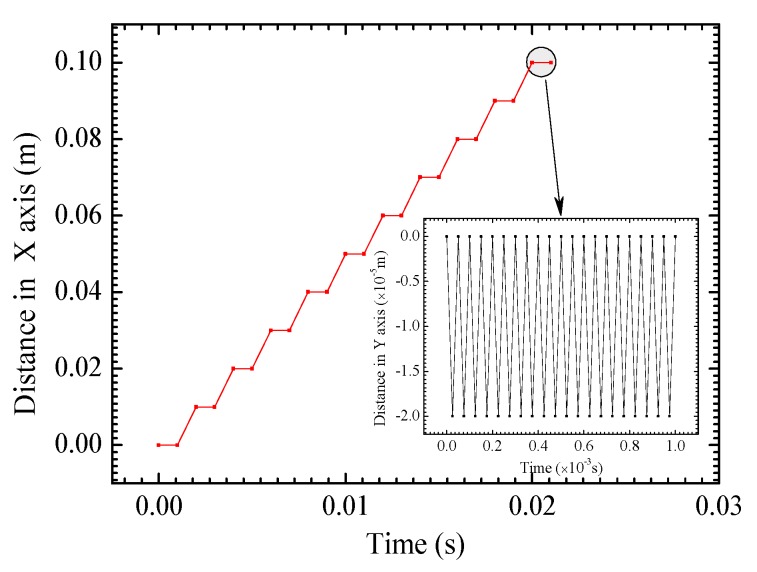
Movement law of pin.

If the UIT process consisting of a large number of impacts is fully analyzed in the simulation, a large amount of time and computation will be required. Hence, it is necessary to simplify the impact process and avoid the meaningless dynamic response. In this study, the movement of the pin controlled by the boundary condition can be decomposed into the vibration perpendicular to the specimen surface and the translation perpendicular to the weld line as illustrated with Figure 7. The pin is set to continuously impact the plate at the same location for 20 times, which would generate sufficient plastic deformation, and reduce welding residual stress effectively. After each ultrasonic impact, the pin is moved with a distance of one radius, which guarantees the enough impact coverage to make the residual stress as homogeneous as possible, and coordinates the proper iteration time. In order to move the pin along the desired trajectory, not only the boundary condition of moving direction but also the freedom of non-moving direction needs to be set in each step. Persistent residual oscillations after UIT could lead to the numerical instability of residual stress field. The introduction of Rayleigh damping consisting of mass proportional damping and stiffness proportional damping is used to achieve energy dissipation, and the principle formula are shown as Equations (13) and (14) [31,32]:

(13) C=αM+βK

(14) $$\xi =\frac{\alpha }{2\omega }+\frac{\beta \omega }{2}$$

where *C*, *M*, and *K* are the damping matrix, the mass matrix, and the stiffness matrix of the UIT model, *α* and *β* are the mass proportional Rayleigh damping and the stiffness proportional Rayleigh damping that need to be set up in the material property, *ξ* and *ω* is the critical damping and the natural frequency for the given model. *ω* is defined by the model shape and the material properties of the model [31,32]:

(15) $$\omega =\frac{\pi }{2T}\sqrt{\frac{E}{\rho }}$$

where *E* and *ρ* are Young modulus and density that represent material properties, *T* is the plate thickness that stands for model shape. In this study, *β* is defined as zero, and *α* is computed by the above relation. The step period of impact vibration is determined by the impact times and the impact frequency. The dynamic step is applied because the UIT process is a high-speed impact process, which would cause a sudden change in stress and deformation within a short time. The data transmission would be difficult in the explicit step if thermal-mechanical coupling calculation is adopted in the previous steps. The implicit step is a good compromise between the computing time and the calculation accuracy for a simple model, so it could be used in this simulation.

## 4. Results and Discussion

### 4.1. Temperature Distribution

**Figure 8:**
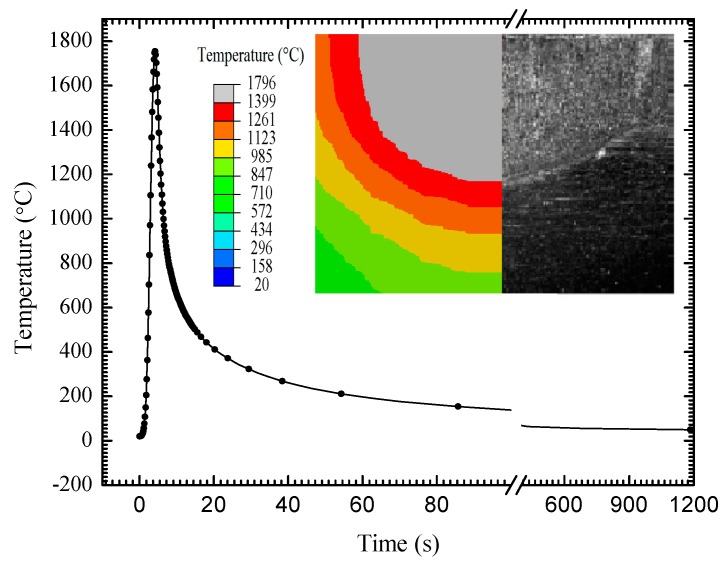
Temperature variation curve and weld pool morphology.

Figure 8 plots the temperature variation curve in the welding process. It shows the characteristics of fast heating and slow cooling, which conforms to the definition principle of the double ellipsoidal heat source distribution model. Figure 7 also compares the temperature contour of weld section by FEM and the weld pool morphology by experiment. It shows that all of the temperatures in the weld region are higher than the material melting point. The simulation result coincides with the experimental result. In the weld zone, the maximum temperature is 1746 °C at the weld center. In the welding heat affected zone (HAZ), the maximum temperature is 1315 °C, which is very close to the weld, and then the temperature decreases gradually away from the weld center.

### 4.2. Residual Stress Distribution before and after UIT

**Figure 9:**
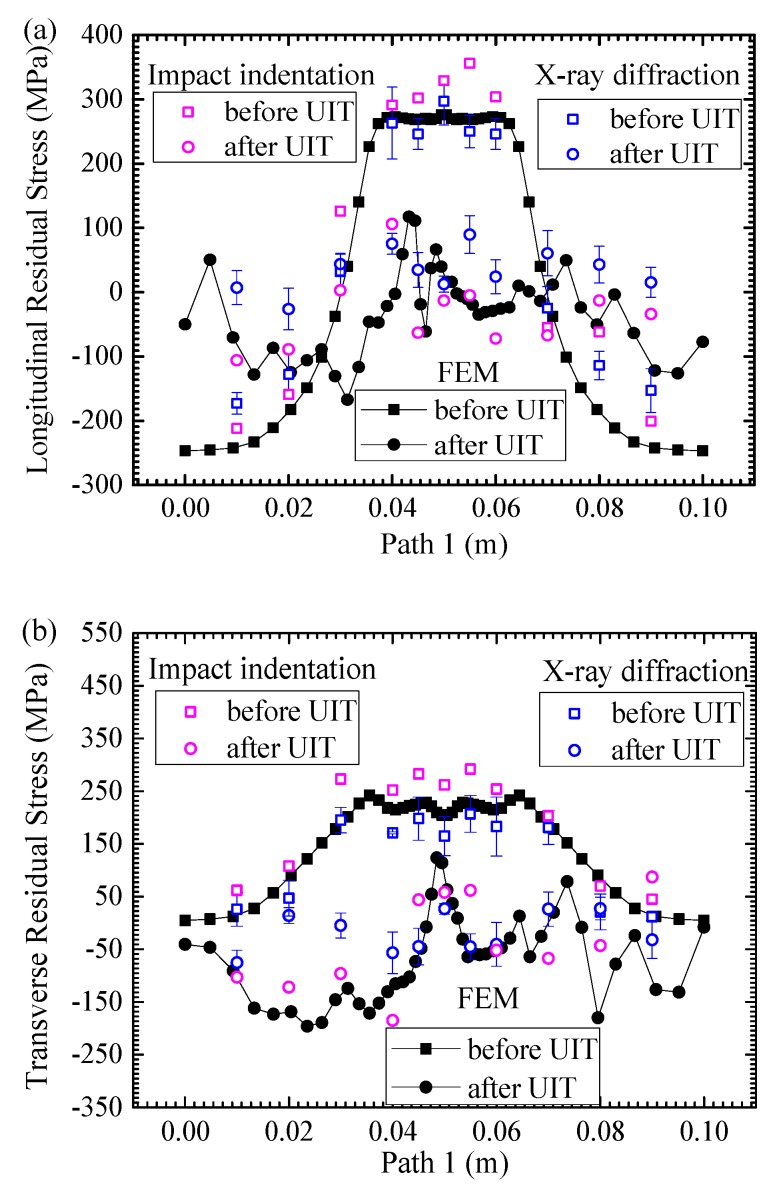
Comparison of longitudinal (**a**) and transverse residual stresses (**b**) before and after UIT by experiment and FEM along Path 1.

From the calculation results, it shows that the longitudinal and transverse residual stresses are larger, and the residual stress along the thickness direction is very small and ignored here. Figure 9 compares the longitudinal and transverse residual stresses along Path 1 (see Figure 1) before and after UIT by simulation and experiment. For the post-weld state, the welding residual stresses are mainly concentrated in the weld region and HAZ. In total, the farther away of location from the weld, the smaller the residual stress. After UIT, the residual stresses are significantly decreased. The residual stresses achieve the status of small tensile stresses and compressive stresses. In the weld region, it can be seen from the simulation results that there is an obvious decrease in the magnitude of the peak and average longitudinal residual stresses from 275.9 and 270.8 MPa to 66.3 and 7.6 MPa, respectively. There is an evident decrease in the peak and average of transverse residual stresses from 228.1 and 218.1 MPa to 123.4 and 24.9 MPa, respectively. In the HAZ, the peak and average of longitudinal residual stresses, with values of 273.2 and 263.3 MPa, are decreased to 117.4 and 1.2 MPa, respectively. The magnitude of peak and average transverse residual stresses decrease from 241.8 and 224.6 MPa to 12.9 and −82.2 MPa, respectively. On the impacted path, most of the longitudinal residual stresses have been in a small tensile stress state, and transverse residual stresses are basically compressive stresses after UIT. It proves that UIT could effectively reduce the welding residual stress.

At the as-welded state, the simulation data is in good agreement with the experimental data in the weld region and HAZ, which shows that the welding simulation is reliable and accurate. There is a discrepancy between simulation and experiment in the base metal, because the as-fabricated stresses are ignored in the calculation. After UIT, in total the simulation results approximately agree well with the measurement results, but still have a difference. The reason is that the load is simplified in the finite element analysis. In addition, the simulation is considered to be ideal, but there are various uncontrollable factors such as uneven impacts in the experiment. It can be clearly seen from the graph that the accuracy of welding simulation results is higher than UIT simulation results. The welding residual stress is generated by thermal deformation with slow response, but the residual stress after UIT is generated by plastic deformation with fast response. The energy output of welding heat source is stable, but the energy output of UIT with high frequency is unstable. The welding direction is along the weld, but the UIT direction is perpendicular to the weld. So, the welding residual stress distribution is symmetric, and the residual stress distribution after UIT does not.

**Figure 10:**
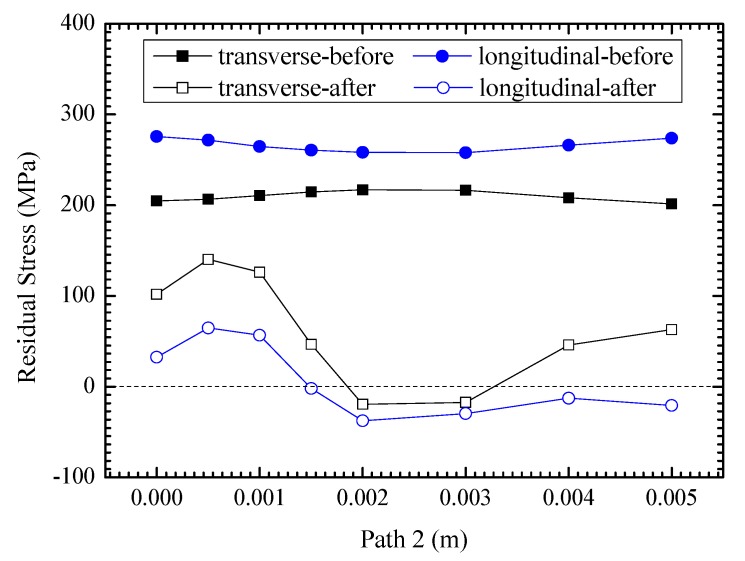
Comparison of the residual stress distributions before and after UIT along Path 2.

**Figure 11:**
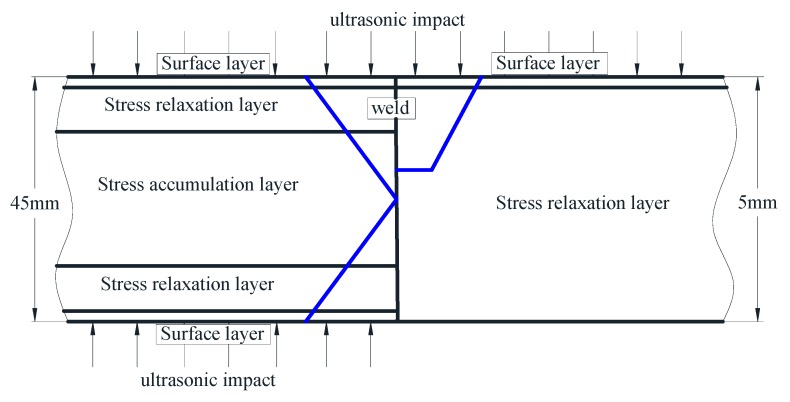
Hierarchical divisions based on the UIT effect under different thicknesses.

As shown in Figure 10, the internal residual stresses, including longitudinal stress and transverse stress, generally decrease greatly after UIT, which demonstrate compressive residual stress states at the middle section of sample. Due to the direct impact of the pin, the residual stress of upper surface is obviously lower than that of the subsurface. In the lower surface area, the longitudinal stress is shown as small compressive stress, and the transverse stress is shown as small tensile stress. The average decrease of longitudinal residual stress is 259.9 MPa, greater than 149.1 MPa of transverse residual stress. The longitudinal tensile residual stress is eliminated at about 1.5 mm below the upper surface, and the transverse tensile residual stress is removed within about 1.9 to 3.3 mm. The hierarchical partition is distinct according to the UIT effect in the thickness direction. Combined with the results of Liu et al. [25], the comparison of residual stress distribution after UIT in the different plate thickness is shown in Figure 11. The left is 45 mm-thick welded plate treated by UIT on the top and bottom surface, and the right is that of 5 mm treated on the top surface. According to the simulation results, the thin plate is roughly divided into two layers after UIT. The surface layer is impacted directly by the pin, which improves the weld toe morphology, reduces the surface roughness, and adjusts the residual stress distribution in the stress concentration area. Based on the previous analysis, mass plastic deformation occurs after UIT, and the residual stresses at different depths are obviously reduced. Therefore, the whole plate is in the stress relaxation layer. Liu et al. [33] had a double-sided impact on the thick plate, so there are surface layers on the upper and lower surfaces. According to the test results, the residual stresses near the surfaces are obviously decreased, and there are stress relaxation layers beneath the upper and lower surface layers. In order to balance the stresses, there is an increase in the residual stress in the middle layer of the thick plate, which is defined as the stress accumulation layer. It is the biggest difference between the thin and thick plate after UIT.

### 4.3. Mechanism of Stress Relaxation

**Figure 12:**
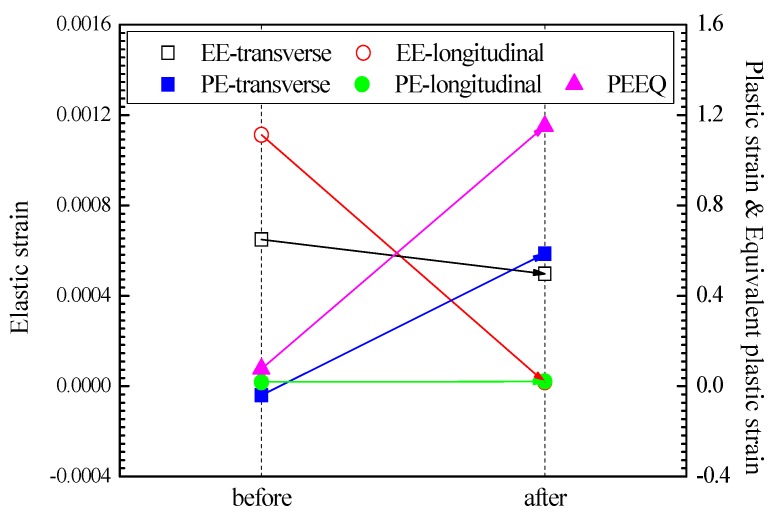
Variation of elastic strain, plastic strain and equivalent plastic strain before and after UIT.

From the macroscopic perspective, UIT could stimulate the stress wave with ultrasonic frequency. Local yield will be produced at the peak of residual stress in the specimen, and the elastic strain will develop to the plastic deformation if the sum of the dynamic stress and the residual stress is greater than the yield strength of the material. As a result, the residual stresses are decreased and homogenized. The changes of elastic strain, plastic strain and equivalent plastic strain before and after UIT at the center point of the welding surface are shown in Figure 12. It can be seen that the elastic strains are slightly decreased and the equivalent plastic strains are markedly increased after UIT, which confirms the above theory. From the view of energy, a large number of metal atoms deviate from the equilibrium in the weldment when free energy is unstable. The principle of minimum energy in thermodynamics shows that the system is always self-adjusting, making the total energy minimized, and making each part in a balanced state. UIT could stimulate the vibration of metal atoms, which accelerates the transformation to the equilibrium position from the higher energy position. Therefore, the total energy of the atom is reduced, the lattice distortion is reduced, and the residual stresses are eliminated. The balance of macroscopic residual stress distribution is maintained by the formation and arrangement of dislocation structure as well as lattice distortion. From the microscopic perspective, the energy is provided by the pin, which breaks the original dislocation structure during the UIT [33,34]. Because the cross-slip of the dislocation is restricted by the low stacking fault energy of austenitic stainless steel, the dislocation movement only occurs on each slip surface, and it may develop to the dislocation block in certain extent. When the internal stress produced by the dislocation block reaches the critical shear stress of the twinning deformation, monophyletic twins will be generated. With the increase of deformation, the twinning deformation gradually transits from single-direction to multi-direction. The grain is crushed under the combined action of twin duplication and twin density increases, which explains the strengthening effect of UIT.

## 5. Conclusions

Experimental and numerical investigations are conducted to study the effect of UIT on reducing the residual stress of the welding. A three-dimensional finite element method, which includes a sequentially coupled thermomechanical welding model and the dynamic elastic-plastic UIT model, is used to calculate the welding temperature field and the residual stress field before and after UIT. The conclusions can be drawn as follows:

(1) The numerical results are in a good agreement with the experimental data, which showed that the UIT could decrease the tensile residual stresses to a large degree and even generate the compressive residual stresses.
(2) After UIT, longitudinal residual stresses basically turned into the small tensile stress state from large tensile stress state, and transverse residual stresses have mainly turned into compressive stresses from large tensile stress.
(3) In the through-thickness direction, the average decrease of longitudinal residual stress is 259.9 MPa after UIT. The longitudinal tensile residual stress is eliminated at about 1.5 mm below the upper surface, and the transverse tensile residual stresses become compressive within about 1.9 to 3.3 mm away from the upper surface.
(4) The overall residual stresses of the thin plate can be sharply decreased after UIT, but the residual stresses are increased in the middle layer of the thick plate.
(5) The plastic deformation, the energy stability and the twinning contribute to the relaxation of residual stresses after UIT.

## Figures and Tables

**Table 1:** Chemical compositions of parent metal and welding wire (wt. %).

Composition	C	Si	Mn	P	S	Cr	Mo	Ni	Cu
304	0.048	0.419	1.228	0.031	0.0018	18.08	0.011	8.113	0.0096
A102	0.053	0.50	1.78	0.028	0.003	18.96	0.26	10.21	0.41

**Table 2:** Main parameters of X-ray diffraction (XRD) measurement.

Parameters	Settings
Target	Mn
Radiation [26]	MnKα-ray
Diffraction lattice plane [26]	{311}
Ψ	0° 22.5° 35° 45°
Stress constant	−289 MPa/°
Scanning angle	148°–158°
Scanning step	0.1°
Counting time	1 s
X-ray tube voltage	20 kV
X-ray tube current	5 mA
Collimator diameter	2 mm
